# DNA Free CRISPR/DCAS9 Based Transcriptional Activation System for *UGT76G1* Gene in *Stevia rebaudiana* Bertoni Protoplasts

**DOI:** 10.3390/plants11182393

**Published:** 2022-09-14

**Authors:** Asish Kumar Ghose, Siti Nor Akmar Abdullah, Muhammad Asyraf Md Hatta, Puteri Edaroyati Megat Wahab

**Affiliations:** 1 Laboratory of Agronomy and Sustainable Crop Protection, Institute of Plantation Studies, Universiti Putra Malaysia, Serdang 43400, Selangor, Malaysia; 2 Biotechnology Division, Bangladesh Sugarcrop Research Institute, Ishurdi, Pabna 6620, Bangladesh; 3 Department of Agriculture Technology, Faculty of Agriculture, Universiti Putra Malaysia, Serdang 43400, Selangor, Malaysia; 4 Department of Crop Science, Faculty of Agriculture, Universiti Putra Malaysia, Serdang 43400, Selangor, Malaysia

**Keywords:** CRISPR, dCas9, VP64, stevia, *UGT76G1*, stevioside, rebaudiosideA, biosynthesis, transcriptional activation, sgRNA, protoplast

## Abstract

The UDP-glycosyltransferase 76G1 (*UGT76G1*) is responsible for the conversion of stevioside to rebaudioside A. Four single guide RNAs (sgRNAs) were designed from the *UGT76G1* proximal promoter region of stevia by using the online-based tool, benchling. The dCas9 fused with VP64 as a transcriptional activation domain (TAD) was produced and purified for the formation of ribonucleoproteins (RNPs) by mixing with the in vitro transcribed sgRNAs. Protoplast yield was the highest from leaf mesophyll of in vitro grown stevia plantlets (3.16 × 10^6^/g of FW) using ES5 (1.25% cellulase R-10 and 0.75% macerozyme R-10). The RNPs were delivered into the isolated protoplasts through the Polyethylene glycol (PEG)-mediated transfection method. The highest endogenous activation of the *UGT76G1* gene was detected at 27.51-fold after 24 h of transfection with RNP30 consisting of CRISPR/dCas9-TAD with sgRNA30 and a similar activation level was obtained using RNP18, RNP33, and RNP34, produced using sgRNA18, sgRNA33, and sgRNA34, respectively. Activation of *UGT76G1* by RNP18 led to a significant increase in the expression of the rate-limiting enzyme *UGT85C2* by 2.37-fold and there was an increasing trend in the expression of *UGT85C2* using RNP30, RNP33, and RNP34. Successful application of CRISPR/dCas9-TAD RNP in activating specific genes can avoid the negative integration effects of introduced DNA in the host genome.

## 1. Introduction

**Figure 1:**
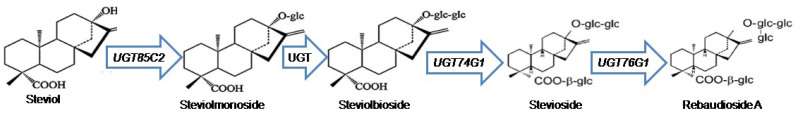
The simplified schematic MEP-pathway for SGs biosynthesis.

Stevia (*Stevia rebaudiana* Bertoni) is cultivated in South and East Asia due to its high dietary and economic potential [1,2]. Botanically, this shrub has vast roots and fragile stems with a few leaves. Steviol glycosides (SGs) found in stevia leaves are extracted to produce non-caloric natural sweeteners [3]. Stevia has been the subject of various biotechnology-based studies including transcriptomics and metabolomics approaches to better understand the SGs biosynthetic route to support its commercial interest [4,5]. Stevioside and rebaudioside A make up the majority of stevia’s SGs [6]. These SGs are synthesized as secondary metabolites and have a taste that is 300-foldsweeter than sugarcane table sugar [7]. They are classified under diterpenoids that exhibit varying degrees of sweetness based on the number and form of sugar groups (glucose, rhamnose, or xylose) replaced on their non-sugar side chains (aglycon) [8]. Richman et al. (2005) discovered three uridine diphosphate glycosyltransferase (UGT) genes (*UGT85C2*, *UGT74G1,* and *UGT76G1*) involved in stevioside and rebaudioside A biosynthesis from *S. rebaudiana* Bertoni expressed sequence tags collection, suggesting that these genes are quite abundantly expressed [9]. The *UGT85C2* converts steviol to steviolmonoside, *UGT74G1* is responsible for the conversion of steviolbioside to stevioside, and in the final step of the MEP-pathway, the *UGT76G1* converts the stevioside to rebaudioside A [10] as shown in Figure 1. The accumulation of rebaudioside A directly correlated with the expression level of *UGT76G1* [11].

The protoplast-based transient expression system (PTES) has proven to be an effective molecular biologicaltool. It is highly adaptable for high throughput and rapid screening for gene functional studies compared to using a transgenic plant system [12,13,14,15]. For protoplast isolation, immature plant tissues such as the hypocotyl, cotyledon, root hair, root, and leaf have typically been employed as source materials [16]. Leaf mesophyll tissues are the most common source of protoplast isolation from different model plants such as *Arabidopsis* [17], rice [16], and tobacco [18], as well as from different non-model plants such as *Panicum virgatum* [19], *Heveabrasiliensis* [20], *Elaeisguinensis* [21], Magnolia [22], and *Phaseolus vulgaris* [23]. Stevia protoplasts were also efficiently isolated from the leaves of in vitro-grown stevia at four weeks of age [24]. In addition to species and explants, a combination of cell wall-degrading enzymes andthe concentration ofD-mannitol frequently used as an osmotic regulator have an impact on protoplast release [21,25]. Protoplasts enable the direct delivery of gene editing tools into cells [26,27,28]. They preserve their cell identity and differentiated condition and, in some plant species, can regenerate into a complete plant.

The clustered regularly interspaced short palindromic repeats (CRISPR)/dCas9 technique has been widely employed in plants to make genomic alterations, providing an effective system for precision agricultural trait improvement [29]. CRISPR/dCas9 DNA constructs are normally transferred into plant cells by standard transformationapproaches such as *Agrobacterium*-mediated transformation [30], particle bombardment [31], and PEG-mediated transformation (for plant protoplasts) [32]. This raises the likelihood of undesirable genetic modifications, the most significant of which are transgene integration and off-target mutation.

The possibility of plasmid or foreign DNA sequences integrating into the host genome can be avoided by employing ribonucleoproteins (RNPs) consisting of preassembled purified dCas9 fusion proteins and in vitro transcribed (IVT) sgRNA. RNPs transferred into protoplasts are not incorporated into the genome of the target host plant and selectivity of the target gene can be performed based on sgRNAs [33]. The Cas9 protein remains in the cell for several days when supplied as a plasmid, however, it is destroyed within 24 h when administered as RNPs, increasing the specificity of the targeting mechanism [34]. Meanwhile, dCas9 is susceptible to protein degradation because of its highersequence repetition. Genetic fusion of XTEN [35], *30Kc19* [36], or other protein-stabilizing polypeptides to the amino or carboxy terminus of dCas9 may mitigate this issue and further enhance the dCas9-mediated transcriptional activation [37]. XTEN has the benefit of being able to function as bridging between protein domains, allowing for multispecificity and multivalency. Moreover, XTEN’s high solubility enhances the stability of various payloads, which could make the production of protein products easier [35].

Plants have been successfully used to demonstrate the applicability of DNA-free CRISPR technology as an alternative solution to transgenic technology [38]. In recent years, successful desired and stable nucleotide alterations with RNPs have been reported in maize [39], potato [40], *Petunia* × *hybrida* [41], apple, and grapevine [42], rice [43], and wheat [31]. In the majority of these studies, PEG-mediated protoplast transfection was used as a superior method of transformation to introduce RNPs into the cells, and plants were regenerated maintaining the inheritance of the altered nucleotides [38,40,44,45]. The regenerated plants grown from protoplasts devoid of foreign DNA integrated into the genome are expected to receive better public acceptance [38], though the biosecurity of genome-modifiedplants is currently a major public issue [46]. The eradication of transgene integration and minor DNA insertions in mutants created by CRISPR/dCas9 RNP-mediated genome editing is the most significant benefit [47]. Plants regenerated from protoplast cells without integration of DNA would most likely evade the regulatory process [48,49].

This study focuses on enhancing the transcriptional activity of *UGT76G1*, the gene responsible for the biosynthesis of SGs in stevia using CRISPR/dCas9 platforms. The objective of this study was to establish an efficient protoplast isolation protocol from stevia and a DNA-free CRISPR/dCas9-based gene activation system using the isolated protoplasts by designing and synthesizing efficient sgRNAs as well as a dCas9-VP64 fusion protein. *UGT76G1* as the target gene is responsible for increasing the sweet taste of stevia while decreasing the bitter taste [50]. For developing a standard protocol, this study evaluated the appropriateness of RNP-mediated transfection in stevia protoplasts for high-efficiency transcriptional activation of a target key gene in the biosynthesis of SGs.

## 2. Results

### 2.1. Optimized sgRNAs Designing and Selection of Efficient sgRNAs

Using the online tool Benchling(www.benchling.com accessed on 16 May 2021), 35 sgRNAs were determined by utilizing the DNA sequence 529 bp upstream of the TSS. Efficient sgRNAs were selected based on their On-Target (specificity) score, Off-Target (efficiency) score, and GC (%) content. The results obtained from on-target and off-target scores range from 14.16 to 93.68 and from 17.18 to 68.29, respectively, which are in the recommended score varying from 0 to 100. The sgRNAs that have higher than 50 on-target and off-target scores were selected. By this method, sgRNA1, 2, 5, 8, 9, 10, 11, 12, 14, 15, 16, 17, 19, 20, 21, 23, 24, 27, 28, 31, and 32 were discarded while sgRNA3, 4, 6, 7, 13, 18, 22, 25, 26, 29, 30, 33, 34, and 35 were selected.

Among the 12 (sgRNA3, 4, 6, 13, 18, 25, 26, 29, 30, 33, 34, and 35) selected sgRNAs, there were two sgRNAs (sgRNA3 and 6) discarded based on alignment statistics of nucleotides performed through blast (https://blast.ncbi.nlm.nih.gov/Blast.cgi accessed on 16 May, 2021). Ten sgRNAs (sgRNA4, 13, 18, 25, 26, 29, 30, 33, 34, and 35) aligned perfectly with stevia *UGT76G1* gene, promoter region, and UTR; of *Accession No.: KM206772.1* showing maximum score (40.1), the total score (40.1), query cover (100%), E-value (0.72), and percent identity (100) having no gaps. On the other hand, the two discarded sgRNAs (sgRNA3 and 6) did not show any alignment with stevia *UGT76G1*.

PAM sequences were considered in the advance selection of sgRNAs which profiles the secondary structure. Most likely, the function of the sgRNAs relies on the interaction of their secondary structure with the dCas9 protein in vivo. A connection between secondary structure and editing effectiveness of sgRNAs for the CRISPR/dCas9 system was reported. The secondary structure of sgRNA may interfere with the editing efficiency [51,52]. A further selection of sgRNAs (sgRNA18, 30, 33, and 34) (green colored) in Table 1 was performed based on the recommended criteria for efficient sgRNAs selection as the total base pairs (TBP) between the guide sequence and the other sequence should not be higher than 11, consecutive base pairs (CBP) not higher than 6 while internal base pairs in the guide sequence (IBP) not greater than 5. The secondary structure and structural features of selected ten sgRNAs were designed by using the online tool; RNAfold web server (http://rna.tbi.univie.ac.at/cgi-bin/RNAWebSuite/RNAfold.cgi accessed on 16 May 2021) [53] (Table 1).

**Figure 2:**
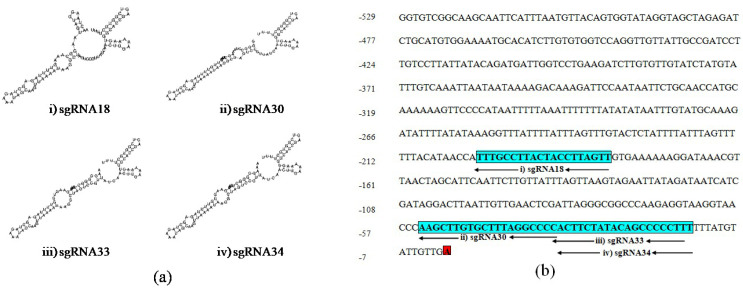
Schematic secondary structure of the selected sgRNAs and their position on the proximal promoter region of *UGT76G1*: (**a**) The secondary structure of sgRNAs: (i) sgRNA18, (ii) sgRNA30, (iii) sgRNA33, and (iv) sgRNA34, and (**b**) Blue boxes show the sequence and positions of four selected sgRNA:; (i) sgRNA18, (ii) sgRNA30, (iii) sgRNA33, and (iv) sgRNA34, and red box shows transcription start site (TSS).

The secondary structure of sgRNAs showed that two out of four of the designed sgRNAs, sgRNA18 and sgRNA30, have intact secondary structures including stem-loop RAR, stem-loop one, stem-loop two and stem-loop three while the rest of the two sgRNAs, sgRNA33 and sgRNA34 have an intact structure including stem-loop RAR, stem-loop one, stem-loop two, stem-loop three and stem-loop four (Figure 2a). Therefore, the advanced sgRNAs selection was narrowed down to four sgRNAs (sgRNA18, sgRNA30, sgRNA33, and sgRNA34) considering all recommended characteristics complementary to the *UGT76G1* target gene sequence and with different lengths at −200, −54, −35, and −34 upstream of the TSS, respectively (Figure 2b).

### 2.2. Assessment of the Effect of Enzyme Solutions on Releasing of Living Protoplast from Stevia

Stevia leaf mesophyll and callus were used to isolate protoplasts. Due to the existence of chlorophyll in the chloroplast of leaves, the protoplasts recovered from stevia leaf mesophyllwere vivid green in color, and protoplasts isolated fromcallus lacking in chlorophyll were light in color. After overnight (16 h) incubation at 25 ± 2 °C, all of the enzyme solution combinations (ES1, ES2, ES3, ES4, ES5, ES6, ES7, ES8, and ES9) were capable of releasing protoplasts from 28 d old leaf mesophyll and callus of stevia. Supplementary Figure S1 illustrates the microscopic view of released protoplasts which were quantified by using a hemocytometer. The results show the effects of different enzyme solutions in releasing protoplasts from stevia leaf mesophyll and callus.

**Figure 3:**
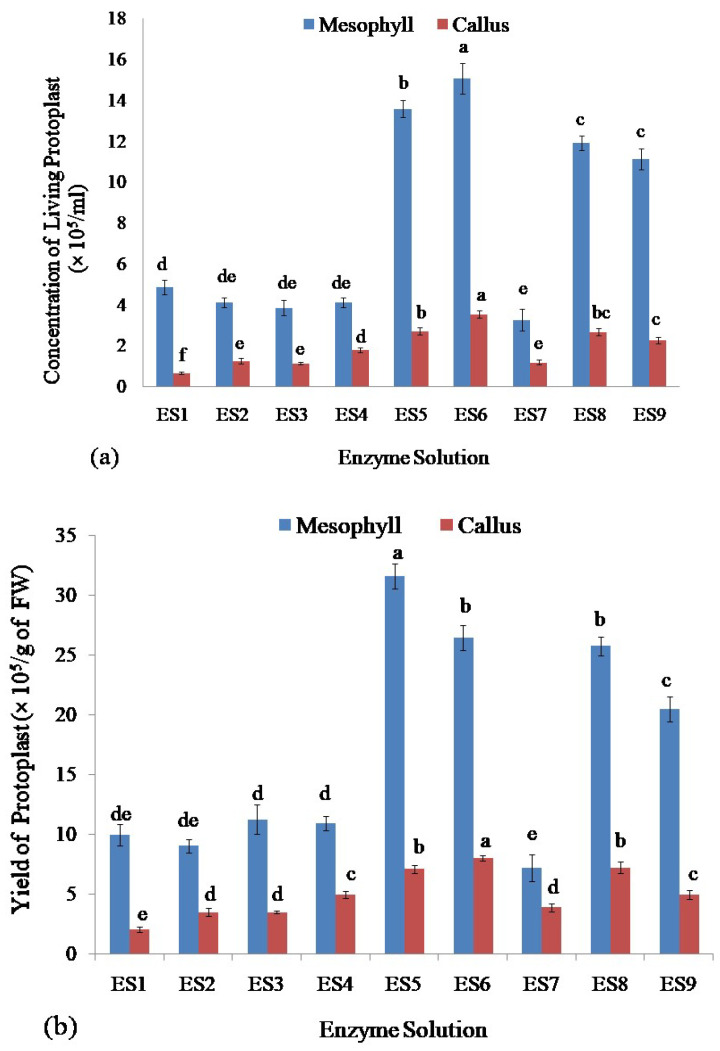

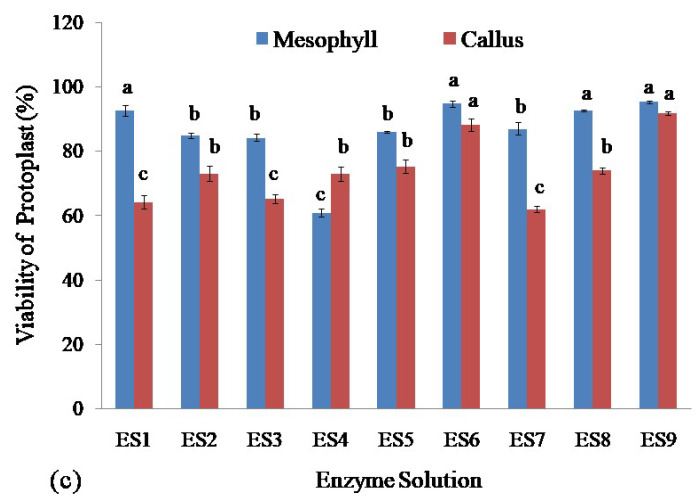
Assessment of protoplasts isolated from leaf mesophyll and callus of stevia by using different concentrations of cellulase R-10 and macerozyme R-10 enzyme solutions: (**a**) Comparison of concentration of isolated living protoplasts; (**b**) Assessment of yield; and (**c**) Viability of protoplasts. Bars denoted the mean of three measurements per treatment ± SE (Standard Error). Mean values with the same letters are not significantly different based on the student’s t-test at *p =* 0.05.

Among all of the enzyme concentrations, ES6 produced the highest concentration of living protoplasts from leaf mesophyll (15.07 × 10^5^/mL) as well as from callus of stevia (3.53 × 10^5^/mL)(Figure 3a) whereasES7 and ES1 concentrations of enzymes released the lowest number of living protoplasts from leaf mesophyll (3.27 × 10^5^/mL) and callus (0.67 × 10^5^/mL), respectively (Figure 3a).

### 2.3. Assessment of Yield and Viability of Protoplasts Isolated from Leaf Mesophyll and Callus of Stevia by Using Different Enzyme Solutions

The viability and yield of protoplasts isolated from stevia were assessed using the FDA by following the complete protocol reported recently by Lin et al. (2018) [54], and Ren et al. (2020) [55]. Assessment of the yield of isolated protoplasts showed that the enzyme solution ES5 gave the highest yield of the protoplast (31.60 × 10^5^/g of FW) from leaf mesophyll and enzyme solution ES6 produced the highest yield of the protoplast (8.00 × 10^5^/g of FW) from callus of stevia (Figure 3b). The lowest yield of protoplast was achieved from the enzyme solution ES7 and ES1 from leaf mesophyll (7.18 × 10^5^/g of FW) and callus (2.00 × 10^5^/g of FW), respectively (Figure 3b).

The viability of protoplast isolated from stevia was assessed. The highest viability was obtained for protoplasts isolated from leaf mesophyll by using enzyme solutions ES1 (92.68%), ES6 (94.88%), ES8 (92.73%), and ES9 (95.46%) and there were no significant differences among them. The lowest viability of the isolated protoplasts from stevia was found using ES4 which was 60.93% (Figure 3c).

The highest viability of protoplasts from callus was found using enzyme solutions ES6 and ES9 which were 88.23% and 91.81% respectively and there was no significant difference between them. On the other hand, the enzyme solutions ES1, ES3, and ES7 gave the lowest efficiency in achieving viable protoplasts isolated from the callus which were 64.22%, 65.28%, and 62.05%, respectively, and also there was no significant difference among them (Figure 3c).

### 2.4. PEG-Mediated Transfection of Protoplast with dCas9-TADs RNP 

Desalted and concentration adjusted dCas9-VP64 transcription activation domain were separately combined with four previously in vitro transcribed sgRNAs, namely sgRNA18, sgRNA30, sgRNA33, and sgRNA34 at a 1:1 ratio targeting *UGT76G1* different proximal promoter positions at −200, −54, −35 and −34, respectively, to form RNP (ribonucleoprotein) complexes. The RNPs were transfected into the protoplast isolated from stevia leaf mesophyll by PEG-mediated transfection to transcriptional activation of the expression of this gene. After transfection, the protoplasts were harvested by centrifugation. The pellets of protoplast were clearly observed at the bottom of the tubes. 

### 2.5. Quality and Concentrations of RNAs Isolated from Transfected Protoplasts of Stevia and cDNAs 

Total RNA was isolated from PEG-mediated transfected protoplasts. As quantified by Nanodrop measurements, the A_260_/A_280_ value for isolated RNAs from control, RNP18 (sgRNA18), RNP30 (sgRNA30), RNP33 (sgRNA33), and RNP34 (sgRNA34) preassembled with dCas9-VP64 ranged between 1.62 and 1.79 before DNase I treatment and increased to between 1.72 and 1.80 after removal of the DNA. The final yield obtained ranged from 10.8 to 44.7 ng/µL (Table 2). The total RNA was then analyzed by electrophoresis on 2% (*w/v*) agarose gel as shown in Supplementary Figure S2a. The distinct bands corresponding to 28S and 18S RNAs were clearly visible showing that the RNA was of good integrity without DNA contamination. Washing of transfected protoplasts with PBS (pH 7.00) allows removal of incubation solution (WI) from the protoplasts, and flash frozen by liquid nitrogen arrests the metabolic activities of the cells and protects the RNA from degradation. The RNAs from different transfected protoplast samples were reverse transcribed to detect the expression of the dCas9-TAD transcriptional activated *UGT76G1* in stevia. The expression of *UGT76G1* in control was observed as a faint PCR product whereas, in the dCas9-TAD gene-activated samples, the bands of the expected size (94 bp) were visible (Supplementary Figure S2b). The visible bands showed the high quality of reverse transcribed cDNAs from RNAs of the transfected protoplast of stevia (Supplementary Figure S2b).

### 2.6. Impact of Different sgRNA Positions on the Transcriptional Activation Level of UGT76G1 in Stevia Protoplast Transfected with Different RNP Complexes

**Figure 4:**
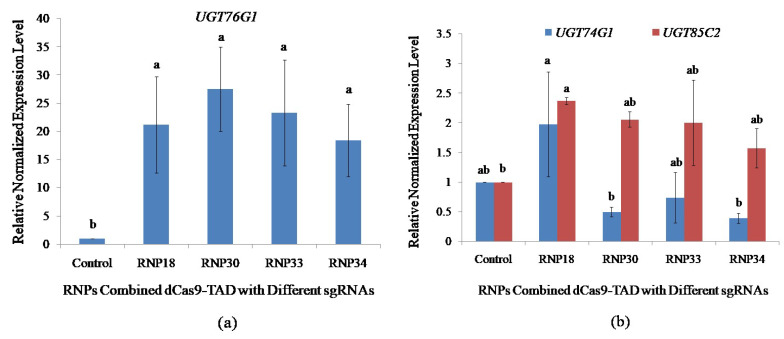
Impact of sgRNA positions on the activation of *UGT76G1* in transfected protoplasts and the effect of *UGT76G1* activation on the expression of other rebaudioside A biosynthetic pathway genes (*UGT74G1* and *UGT85C2*). qPCR was performed on the stevia protoplast transfected with the different RNPs combination of dCas9-VP64 with sgRNAs targeting different proximal promoter positions of *UGT76G1*. (**a**) Positional impacts of sgRNAs on transcriptional activation level of *UGT76G1*; and (**b**) Effects of activating *UGT76G1* on the expression of other key biosynthetic pathway genes. The expression was normalized with three reference genes; *Actin, Aquaporin*, and *Calmodulin*. Bars denoted the mean of three biological replications per treatment ± SE (Standard Error). Mean values with the same letters are not significantly different based on the student’s t-test at *p =* 0.05.

In order to quantify the level of gene activation, quantitative polymerase chain reaction (qPCR) analysis was conducted using cDNA synthesized from RNA extracted from the protoplasts transfected with RNP complexes containing different sgRNAs. The four antisense sgRNA molecules were designed based on all of the parameters with variable distances from the TSS. The in vitro transcribed sgRNA18 (RNP18), sgRNA30 (RNP30), sgRNA33 (RNP33), and sgRNA34 (RN34) were at positions −200 bp, −54 bp, −35 bp, −34 bp upstream of the TSS, respectively. Induction of *UGT76G1* expression was successful using all the RNPs with enhancement expression ranging from 18.39- to 27.51-fold identified after 24 h of activation (Figure 4a). The RNP30 had the greatest endogenous gene expression, showing an increase of 27.51-fold for *UGT76G1*, but RNP18, RNP33, and RNP34 showed a moderate level of activation of 21.17, 23.27, and 18.39-fold, respectively. Transfection without RNP was unable to activate the *UGT76G1* target gene. The qPCR analysis revealed a significant increase in expression driven by the dCas9-TAD transcriptional activator compared with the control. However, there was no significant difference in the activation level among all four sgRNAs at *p=0.5* using the student’s t-test.

### 2.7. Effect on the Expression Level of Other Key UGTs Involved in SG Biosynthesis Pathway by Transcriptional Activation of UGT76G1 in Stevia Protoplasts

The effects on the expression of other key *UGT* genes involved in rebaudioside A biosynthesis due to the activation of *UGT76G1* expression in stevia protoplasts were analyzed by qPCR. This will provide information on the transcriptional regulatory network influenced by the transcriptional regulation of *UGT76G1* through stevia protoplast cell-based assay. An increase in *UGT76G1* expression driven by dCas9-VP64 RNPs did not result in a significant change in expression of *UGT74G1* in protoplasts transfected with RNP18, RNP30, RNP33, and RNP34 at *p =* 0.05 using student’s t-test (Figure 4b). 

The highest change in the expression level of *UGT85C2* was found utilizing RNP18 which showed a significant increase in expression by 2.37-fold. The RNP30, RNP33, and RNP34 showed an increasing trend in the expression level of *UGT85C2* by 2.06-, 2.00-, and 1.57-fold, respectively, even though not significantly different at *p =* 0.05 compared to transfecting the sample without RNP (Figure 4b). This suggests that the increase in the expression of *UGT76G1* leads to an increase in the expression of *UGT85C2* at the earlier step of the biosynthetic pathway, possibly to channel more intermediates to meet the increase in the production/activity of *UGT76G1*.

## 3. Discussion

The CRISPR/dCas9 technology was employed to enhance the transcriptional activity of the *UGT7G1* gene in stevia. Activation of the target gene was achieved using RNP complexes formed by combining different sgRNAs in vitrotranscribed from variable locations in the proximal promoter regions of this gene and pre-synthesized dCas9-VP64 protein. The enhancement in the transcriptional activity was evaluated based on the expression level of the target gene following the transfection of stevia protoplasts with the different RNP complexes.

Plant protoplasts from different plant species have been employed for characterizing gene functions, particularly the roles of different genes in signaling and biosynthetic pathways [42]. Transient expression methods using protoplasts from model plants such as *Arabidopsis* [56] and rice [57] were used for investigating gene activity, protein subcellular distribution, and protein-protein interactions. The approach presented here minimizes the amount of time and source material needed to isolate stevia protoplasts, and it was integrated into a transient gene expression system developed for stevia.

The protocol is faster than the protoplast isolation method reported by Zhao et al. (2011) from pineapple [58], and Kang et al. (2020) from *Petunia × hybrida* Cv. Mirage rose [59] because it does not require a pre-plasmolysis step which usually takes about 1.5 h and decreases the initial plant material used, typically between 2.0 g to 3.0 g [22,56,58] to 0.50 g. Protoplast transient expression methods rely on a large output of viable protoplasts from healthy plants [60]. Due to the low fiber content, young plants produced in vitro are more appropriate for protoplast isolation than older field-grown plants for releasing a higher number of protoplasts [61]. In the present study, in vitro-grown stevia plantlets were used for protoplast isolation. Protoplasts lacked cell walls, which regulate the exterior and interior cellular environments to help in maintaining the osmotic gradient [58,62]. even without cell walls, the correct osmolarity is essential for the protoplast to exist. In the current investigation, the concentration of mannitol as the osmoticum regulator used for the separation of stevia protoplast was 0.4 M following the reports by Jia et al. (2016) [63], Li et al. (2018) [64] and Cheng and Nakata (2020) [65]. In addition, sharp razor blades were used to prevent mechanical damage and edge crushing when preparing the plant samples for digestion with cell wall-degrading enzymes.

Analysis using nine different concentrations of cellulase R-10 and macerozyme R-10 showed that the protoplast yield was the highest from leaf mesophyll of in vitro-grown stevia plantlets (3.16 × 10^6^/g of FW) using ES5 (1.25% cellulase R-10 and 0.75% macerozyme R-10) and highest from stevia callus (8.00 × 10^5^/g of FW) using ES6 (1.25% cellulase R-10 and 1.00% macerozyme R-10). The yield from leaf mesophyll is much higher than those reported from other plant species such as pineapple [66], *Phaseolus vulgaris* [23], and *Petunia × hybrida* Cv. Mirage rose [59], but lower than the previously isolated protoplast from stevia reported by Lopez-Arellano et al. (2015) [24] which utilized DriselasePectolyase Y-23 as the components of enzyme solution, and 0.5 M mannitol. The advantage of including callus in our study was that the starting material can continuously be produced and maintained in culture. Our results reveal that the concentration of the cell wall-degrading enzymes affected protoplast yield and the source tissues for protoplast isolation influenced the release of viable protoplasts consistent with previous reports [16,67,68]

The CRISPR/dCas9 system is a platform for precise genetic control of genomic targets. CRISPR/dCas9 as a re-engineering platform for regulating gene expression in plants offers several benefits including simplicity, target specificity, flexibility, and reversibility [69]. dCas9 activation systems use transcriptional activator(s) at the protein’s N or C terminus [37,70] to control the transcription of endogenous genes without irreversible alteration of the genome. So far, there are limited reports on functional genetic activation in plants using CRISPR/dCas9 platforms [37,71,72] and the majority of studies have focused on stable transformation involving *A. tumefaciens.* A very robust CRISPRa system developed by Pan et al. (2021) as CRISPR–Act3.0 works in tomato, rice, and *Arabidopsis* by methodically testing multiple effector recruitment tactics and transcription activators based on deactivated *Streptococcus pyogenes* dCas9 (dSpCas9). It achieved four to six times the activation of current CRISPRa systems [73]. While the dCasEV2.1 activation mechanism in plants is a powerful CRISPR tool for gene induction, with enhancement in genome-wide specificity paired with a high activation capacity [74]. To the best of our knowledge, our study is the first reporting on a successful demonstration of CRISPR/dCas9 gene activation in stevia protoplasts via RNPs-mediated protoplast transfection.

VP16 is involved in multiple interactions with components of eukaryotic basal transcriptional machinery including with the different general transcription factors and mediator complex [75] to facilitate in the assembly of the pre-initiation complex [76]. Several investigations have demonstrated that dCas9-VP64, which employs the VP16 tetramer is effective in activating gene transcription [37,68,71,72] and recent research has shown that the dCas9-VP64 may be employed in plants to activate endogenous genes [69]. In the present study, the effectiveness of targeted gene activation in plant cells was evaluated using different combinations of purified dCas9-VP64 protein and IVT-sgRNAs targeting specific regions of the *UGT76G1* gene inthe stevia that make up the RNP complexes. PEG-mediated transfection introduced the RNP complexes into the protoplasts of stevia. *UGT76G1* was strongly activated as a target gene by 18.39-fold to 27.51-fold, as measured by qPCR 24 h after transfection. This is higher than what has been achieved by previous researchers using the dCas9-VP64 gene activation system. Li et al. (2017) found that 5 h after PEG-mediated transfection, dCas9-VP64 RNP could activate *ArabidopsisWRKY30* by 11.7-fold, rice *ER1* by 13-fold, and *ArabidopsisRLP23* by 9.3-fold [37]. Therefore, future analysis at different time points post-infection is suggested to see whether it influences the activation level.

sgRNAs-guided dCas9 activators that boost the transcriptional activity of the target gene serve a critical role in CRISPR gene activation [77]. In the present study, four sgRNA molecules, complementary to the promoter sequences and with variable distances from the TSS were generated and in vitrotranscribed. The sgRNA18, sgRNA30, sgRNA33, and sgRNA34 were positioned at −200, −54, −35, and −34 upstream of the TSS, respectively. All the sgRNAs target the sense strands of the genomic sequences. The sgRNAs were selected based on several important characteristics that influence binding and transcriptional activity. These include on-target and off-target scores [70,71], GC (%) content [77], similarity to the target sequence and secondary structure; (the total base pairs (TBP) involving base-pairing between guide sequence and the other sequence, consecutive base pairs (CBP), and internal base pairs (IBP) in the guide sequence) [51,52]. The online tool Benchling [78] was used in selecting the sgRNAs based on these criteria.

The positional impacts of sgRNAs in the promoter region on the transcriptional activation efficiency of our target gene, *UGT76G1*, were investigated. Even though all four sgRNAs achieved a high level of transcriptional activation, there is no significant difference in the transcription activation level achieved among them. Our results are consistent with Piatek et al. (2015) where sgRNAs targeting the promoter region 200 bp upstream of the TSS are highly associated withdCas9-TADS-mediated gene activation [72]. The locations of all designed sgRNAs positioned in the proximal promoter region are highly suited to effective recruitment of the basal transcriptional machinery for transcriptional activation.

Steviol glycosylation by the *UGTs* in stevia produces a variety of steviol glycosides with different organoleptic characteristics [79,80]. Studies on functional specificities [81] and transcriptional regulation of *UGTs* [82] provided the essential background knowledge for genetic and metabolic engineering to manipulate the biosynthetic pathway to improve steviol glycoside output. Overexpression of *UGT76G1* in transgenic stevia led to altered composition of SGs specifically enhancing rebaudioside A biosynthesis [83]. The present study explored the effect of induced overexpression of *UGT76G1* on the other two *UGT* genes, *UGT74G1* and *UGT85C2* involved in the biosynthesis of rebaudioside A.

The induced overexpression of *UGT76G1* utilizing different RNPs did not show any significant influence on the expression of *UGT74G1* whereas the *UGT85C2* showed a significant increase in expression (2.37-fold) compared to the control when sgRNA18 was utilized for the overexpression of *UGT76G1*. The effect of using sgRNA30, sgRNA33, and sgRNA34 for activating *UGT76G1* suggests an increase in the expression of *UGT85C2,* even though not significantly different compared to control. Persistent overexpression of *UGT76G1* by gene activation through the dCas9 activator system was predicted in the stevia protoplasts. The concomitant increase in the expression of *UGT85C2* when *UGT76G1* was transcriptionally activated as observed in sgRNA18 potentially allows enough intermediates to be channeled to support the increase in the activity of *UGT76G1* encoded enzyme at the end of the biosynthetic pathway. *UGT85C2* was suggested as a rate-limiting enzyme for SG production. Mohamed et al. (2011) showed an increase in the transcriptional level of *UGT85C2* in stevia correlated with increased production of SGs [84], while Yoneda et al. (2018) reported that induced upregulation of *UGT85C2* by plant growth regulators increased the production of stevioside and rebaudioside A [85]. This suggests that an increase in the expression of *UGT76G1* observed in the present study, which potentially led to an increase in the expression of *UGT85C2*, may elevate production of SGs.

The findings from this study on gene activation by CRISPR/dCas9-TAD RNP recruitment in the plant protoplast to target the gene of interest with greater precision and efficiency may aid plant researchers in integrating gene functions and modifying biological features. From tissue preparation, followed by confocal laser scanning microscopic inspection of the isolated protoplasts until qPCR expression analysis of the target gene for activation, the PTES took less than 24 h. However, further research on the regeneration of gene-activated protoplasts is required for generating DNA-free genetically improved stevia.

## 4. Materials and Methods

### 4.1. PCR Amplification of UGT76G1 and Sequencing

The genomic DNA was isolated from the stevia leaves collected from 30 d-old plantlets grown on MS media without any plant growth hormones. The method for DNA isolation was adopted by Arif et al. (2010) [86]. The *UGT76G1* was amplified by PCR using gene-specific primers (*UGT76G1F*: CCCTACTTGCTACATTCG and *UGT76G1R*: CTGACGTTTACACGCAAG). The primers were designed based on the *UGT76G1* (*Accession No.: KM206772.1*) gene promoter sequence. The PCR was performed in a 50µLreaction mixture containing 4 µL of genomic DNA (50 ng/µL); 25 µL of master mix (BIOLINE, London, UK); 2 µL of each primer of 10 µM concentration, and 17 µL of nuclease-free water. The PCR product was electrophoresed on 0.8% agarose gel containing 1% florosafe (1st BASE, Catalog Number: BIO-5170) for 80 min at 60V (Supplementary Figure S3a). The agarose gel containing amplified *UGT76G1* was purified using QIAquick Gel Extraction Kit (QIAGEN, Catalog Number. 28706) following the manufacturer’s protocol. The eluted DNA was quantified by using Thermo Scientific NanoDrop^TM^ 1000 Light Spectrophotometer (Nanodrop Technologies, Inc, 3411 Silverside Rd, Bancroft Building, Wilmington, DE, USA). The Purified PCR product was sent to Integrated DNA Technologies, Malaysia for sequencing.

The sequences derived from PCR amplified products for both forward and reverse primers were analyzed to generate the complete sequence (Supplementary Figure S3b). A nucleotide sequence of 1839 bp was generated by using the online tool https://www.geneious.com accessed on 20 April 2021 and used to produce the consensus of the *UGT76G1* of 1839 bp. The generated consensus was aligned to determine the similarity with the reference sequence of *UGT76G1* (*Accession No.: KM206772.1*) found in GenBank through an online tool https://www.ncbi.nlm.nih.gov/, accessed on 25 April 2021. and consistent with the findings of Yang et al., (2015) [87]. The alignment showed 99% similarity (1824/1839) with a 0% (6/1839) gap and the annotated sequence covered the range of 99 to 1932 bp within 1994 bp of the *KM206772.1*.

### 4.2. Finding and Retrieving Promoter Sequence from Sequenced Data of the UGT76G1 for Potential CRISPR Targets and Designing of Single Guide RNA (sgRNA)

For designing efficient sgRNAs, it is important to predict the exact location of the transcription start site (TSS). The sequenced data of the stevia *UGT76G1* gene in FASTA format were analyzed by www.softberry.com, accessed on 27 April, 2021 and promoter sequence information such as TSS and TATA box was retrieved. The Benchling (www.benchling.com, accessed on 16 May 2021) online tool for designing sgRNAs was employed as it applies to species such as stevia which has no genomic database. This tool can utilize the nucleotide raw data in FASTA format. The sequence of the upstream region of the transcription start site (TSS) including the TATA box of the promoter region was used in designing.

Softberry identified the nucleotide A (at position +1) as TSS at the nucleotide position 530 of the sequenced region (Supplementary Figure S3b). The TATAbox is at position −33 upstream of the TSS which is located at nucleotide position 497 on the annotated sequence. This also indicates that the *UGT76G1* belongs to the TATA-containing gene. TATA box is an important core promoter element involved in the transcription of eukaryotic genes [88]. This revealed that the *UGT76G1* has a medium-length 5′ UTR of about 1300 bp. In plants,5′*UTR* can even be longer than 2000 bp and it has been suggested that besides regulating gene expression, *UTRs* may contribute to the functional specificity of the genes in a length-based manner [89].

### 4.3. Designing of Single Guide RNA-DNA Template

**Figure 5:**
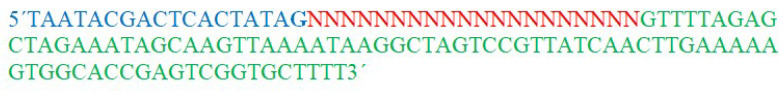
The sgRNA-DNA template sequence. The blue color represents the T7 primer sequence, the red color represents Ns to be replaced by the sequences of different sgRNAs targeting *UGT76G1,* and the green color represents crRNA/crtraRNA 80-nt constant region.

The sgRNAs-DNA template sequences were designed after identifying the target sequence in the promoter region of the *UGT76G1*. The T7 promoter sequence, the sequence coding for target-specific sgRNAs, and the constant region of crRNA/tracrRNA make up the sgRNA-DNA template sequence. The T7 promoter sequence is depicted in blue color in Figure 5. The bold G from the T7 promoter region is included in the transcription. The target region is indicated by red-colored Ns with a length of up to 20 bases. It was notedthat using only 18 bases (deleting the initialtwo bases from the 5′end) can increase specificity [51]. The crRNA/tracrRNA 80-ntconstant sequence is depicted in green (Figure 5).

The NNNNNs in Supplementary Figure S4a was replaced with the target sequences in the selected sgRNAs-DNA template for *UGT76G1* sequences. Out of four sgRNAs, only sgRNA30 naturally had guanine (G) at the 5′ end of the sequence and the remaining three sgRNAs had G at the 5′ end of the sequence from the T7 primer.

### 4.4. Designing of Forward and Reverse Oligonucleotides for PCR Assembly

After fixing the final target sequence, to construct the sgRNAs DNA template, the forward and reverse oligonucleotides were to be Polymerase Chain Reaction (PCR) combined with the Tracr Fragment + T7 Primer Mix supplied in the kit (GeneArt^TM^ Precision gRNA Synthesis Kit, Catalog Number: A29377, Invitrogen, Thermo Fisher SCIENTIFIC, Waltham, MA, USA). The universal forward and reverse amplifying primers, as well as the 80-nt constant region of the crRNA/tracrRNA, are included in the Tracr Fragment + T7 Primer Mix. The synthetic sgRNA primer that overlaps with the Target R1 primer and the 5′ end of the crRNA/tracrRNA constant template requires two 34 to 38 bp oligonucleotides: a Target F1 forward primer with the T7 promoter sequence and a Target R1 reverse primer sequence. Shorter lengths of oligonucleotide (≤40 bases) are advised for target primers to reduce the likelihood of synthesis errors, which are more common with lengthy oligonucleotides. The GeneArt^TM^ CRISPR Search and Design tool provides 34-nt long forward and reverse target primer sequences by default. Having at least one G at the start of the transcript improves sgRNA yield from the in vitro transcription reaction [51]. A 5’ G was added to the target sequence at the T7 forward primer in the Tracr Fragment + T7 Primer Mix used for the sgRNA template assembly. Target region with the added 5’ Gs longer than 21 bases can significantly affect the on-target activity [90]. As transcription starts immediately after the TATA of the T7 promoter sequence, selection of the target sequence was performed by adding one to two 5’ Gs within the 20 bases sequence naturally or using the T7 promoter sequences to have a single G at the 5’ end of the target sequence because it was found to enhance promoter activation by boosting the transcription initiation of sgRNA [88].

### 4.5. PCR Assembly and In Vitro Transcription of Single Guide RNAs (sgRNAs)

The sgRNAs were transcribed in vitro utilizing GeneArt^TM^ Precision gRNA Synthesis Kit (Invitrogen,-Thermo Fisher SCIENTIFIC, Waltham, MA, USA, Catalog Number: A29377) according to the producer’s protocol. PCR-assembled sgRNA DNA template was first performed. Gently, the 10 µM target oligonucleotide mix stock solution was diluted in nuclease-free water to prepare an ordered target oligonucleotide mix working solution (0.3 µM). After that, the PCR assembly reaction was set up as follows: 12.5 µL of Pfusion^TM^High-Fidelity PCR Master Mix(2X), 1 µL of Tracr Fragment + T7 Primer Mix, 1 µL of 0.3 µM Target F1/R1 oligonucleotide mix and 10.5 µL of Nuclease-free water. Then, assembly PCR was performed using the cycling parameters as follows: One initial denaturation cycle at 98 °C for 10 s, followed by 32 cycles of denaturation at 98 °C for 5 s and annealing at 55 °C for 15 s, followed by one final extension cycle at 72 °Cfor one minute, and at last held at 4 °C.The quality of PCR products was analyzed using gel electrophoresis as shown in Supplementary Figure S4b. After PCR assembly of the sgRNA DNA template, in vitro transcription was carried out by setting up the following in vitrotranscription reaction. The components of the reaction were added in the order given as follows: 8 µL of NTP mix (100 mM each of ATP, GTP, CTP, UTP), 6 µL of NTP mix (100 mM each of ATP, GTP, CTP, UTP) gRNA DNA template (from PCR assembly), 4 µL of 5X TranscriptAid^TM^ Reaction Buffer and 2 µL of TranscriptAid^TM^Enzyme Mix. The in vitro transcription reaction was incubated at 37 °C for 4 h in a thermalcycler apparatus. Then, one µL of DNase I was added to the reaction mix after the transcription reaction and incubated again in a thermal cycler apparatus at 37 °C for 15 min. After in vitro transcription was completed, IVT sgRNAs were purified by following (Invitrogen, Catalog Number: A29377) manufacturer instructions by adjusting the volume to 200µLby adding nuclease-free water to the IVT reaction. The concentrations of the purified sgRNAs were determined by using Thermo Scientific NanoDrop^TM^ 1000 Lite Spectrophotometer. Spectrophotometric analysis showed the A_260_/A_280_ values for sgRNA18, sgRNA30, sgRNA33, and sgRNA34 were 1.91, 1.90, 2.00, and 1.95 and the concentrations of sgRNAs were 3.22, 3.66, 4.34, and 2.54 µg/µL, respectively. The synthesized sgRNAs were analyzed by gel electrophoresis and discreet bands at 100 bases indicating that sgRNAs were intact without any contamination, and degradation (Supplementary Figure S4c).

### 4.6. Designing of CRISPR/dCas9- Based Transcriptional Activator (dCas9-VP64)

Invitrogen GeneArt Gene Synthesis custom synthesized fragments for dCas9 C-terminus in-frame fusions of VP64 to construct dCas9 fusions with transcriptional activators. The pET-dCas9-VP64-6xHis was purchased from AddGene (Catalog Number: 62935). Cas9 from *Streptococcus pyogenes* was modified to develop dCas9. The dCas9-VP64 activator was developed by fusing a VP64 Tad to the C-terminus of an existing dCas9 with a few tweaks. Novagen’s pET29 served as the vector backbone for pET-dCas9-VP64-6xHis. His vector was 9544 bp in lengthand the insert was 5161 bplong (Supplementary Figure S5a). The vector was designed for bacterial overexpression and contains a gene forampicillin resistance.

### 4.7. Plasmid Purification, Synthesis, and Construction of CRISPR/dCas9-Based Transcriptional Activators

The recombinant plasmid was purified from a single colony bearing pET-dCas9-VP64-6xHis, which was cultured overnight in LB broth containing ampicillin (100 µg/mL). QIAprep^®^ Miniprep plasmid purification Kit (Qiagen, 19300 Germantown Road, Germantown, MD, USA) was used to purify plasmid DNA from pET-dCas9-VP64-6xHis according to the producer’s instructions. Artificial oligonucleotides (for transcriptional activators) or PCR (for dCas9)products were used to construct the synthetic genes. pET-dCas-VP64-AF595 was used to introduce the fragments. The concentration of plasmid DNAs isolated from transformed bacteria was evaluated using a Thermo Scientific NanoDrop^TM^ 1000 Lite Spectrophotometer. Based on its A_260_/A_280_ ratio of 1.8 and a single intact band detected on an agarose gel, the plasmid was found to begood inquality and free of protein andRNA contamination. The concentration of plasmid DNAwas found as 2191.2 ng/µL. To confirm the final construct, sequencing was done. Within the insertion locations, the sequence identity was 100%.

### 4.8. Recombinant Protein Expression of pET-dCas9-TADs-6xHis Transcriptional Activators

#### 4.8.1. Transformation of pET-dCas9-TADs-6xHis Plasmids

Rosetta (DE3) *E. coli* competent cells were transformed according to the manufacturer’s procedure (Novagen^®^). One tube of the competent cell was withdrawn from the freezer and placed on ice, where it was gently stirred to assure that the cells were evenly suspended. The cells were allowed to defrost for 2–5min on ice. The needed number of polypropylene microcentrifuge tubes (1.5 mL) were then placed on ice and pre-chilled. Twenty microliters of cell aliquots were pipetted into pre-chilled tubes. One microlitre of pET-dCas9-TADs pure plasmid DNA (1–10 ng/L plasmids) was directly introduced to the cells and gently mixed by stirring and kept on ice for 5 min. After that, in a 42 °C water bath, the tubes were heated for exactly 30 s without shaking them and the tubes were kept on ice for two minutes. After that, each tube was filled with 80 µL of SOC medium (RT). Before plating on selective media, the cells were incubated for 60 min at 37 °C with 250 rpm shaking. For the plasmid-encoded drug resistance, transformants were selected by plating on LB media supplemented with Ampicillin (100 µg/mL) and Chloramphenicol (34 µg/mL) antibiotics.

#### 4.8.2. Expression of pET-dCas9-TADs-6xHis Proteins

Rosetta (DE3) cells were transformed with recombinant pET-dCas9-VP64 and cultured overnight on LB/Ampicillin/Chloramphenicol agar plates. A single differentiated colony was recovered on the next day and inoculated in 5 mLLB/Ampicillin/Chloramphenicol broth before being kept in a shaking incubator at 150× *g* at 37 °C overnight. On the third day, 1 mL of overnight cultures was poured into a 250 mL flask containing 100 mL of new 2YT/Ampicillin/Chloramphenicol culture (sterile). By taking 1 mLof blank 2YT/Ampicillin/Chloramphenicol culture, the start reading of OD_600_ was determined. Every 30 min, the OD_600_ of the flasks was measured in a shaking incubator. When the OD_600_ became 0.6 to 0.8, each flask was filled with 0.5 mM IPTG and incubated at 18 °C for the following 16 h.

#### 4.8.3. Isolation of pET-dCas9-TADs-6His Proteins

The pET-dCas9-VP64-6xHIs, a His-tagged dCas9-TADs protein, was successfully produced in Rosetta (DE3) as an *E. coli* BL21 cell strain and extracted by utilizing B-PER^®^ Complete Bacterial Extraction Reagent (Thermo SCIENTIFIC, Waltham, MA, USA, Catalog Number: 89822) according to manufacturer’s protocol. That recombinant protein was collected in pellet form from −80 °C refrigerator. After taking it away, the pellet was weighted out and 5 mL of B-PER^®^ for each gram of pellet was measured and kept at room temperature to warm up. Two microliter 0.5 mM EDTA and 10 µL of protease inhibitor for each ml of B-PER^®^ were added to the B-PER^®^ solution. By warming up, the B-PER^®^ containing EDTA and protease inhibitor were mixed with pellet and dissolved by gentle pipetting and the suspension was incubated for 45 min at room temperature with gentle rocking. To separate the soluble protein from insoluble proteins, centrifugation was performed at 15,000× *g* for 20 min at 4 °C temperature. After centrifugation, the supernatants were collected without disturbing the pellets and passed through 0.45 µm filter by using a sterile syringe and kept in a new tube.

#### 4.8.4. Purification, Desalting, and Concentrating of pET-dCas9-TADs-6His Proteins 

Purification of pET-dCas9-TADs-6His recombinant proteins was accomplished by using Ni-NTA Purification System (Thermo SCIENTIFIC, Catalog Number: 89822) following the manufacturer’s protocol. The purified dCas9-VP64 protein concentrations were 1.11, 0.237, 0.080, 0.050, 0.032, 0.034, 0.033 and 0.069 mg/mL measured by followingbovine serum albumin (BSA) E1% = 6.7 assay using Thermo Scientific NanoDrop^TM^ 1000 Lite Spectrophotometer. The purified recombinant proteins were desalted and concentrated by using Amicon^®^Ultra-15 Centrifugal Filter Devices. Before using the device, it was pre-rinsed twice with Milli-Q^®^ water. The eluted proteins (8 mL) were diluted to 12 mLby adding Milli-Q^®^ water and placed on the filter and centrifuged for 30 min at 5000× *g* speed. Approximately 200 µL of proteins are left over in the filter. A volume of 11.8 mLof dCas9 storage buffer (10% (*v*/*v*) glycerol, 150 mM KCl, 20 mM HEPES (pH 7.5), and 1 mM DTT) was added to the filter and centrifuged for 30 min at 5000×*g* speed following the procedure revealed by Li et al. (2017) [37]. Finally, around 200 µL of desalted protein was recovered from the filter, and concentration was measured by using Thermo Scientific NanoDrop^TM^ 1000 Lite Spectrophotometer. The concentration of dCas9 was adjusted to 500 µg/mL by using dCas9 storage buffer and kept at −80 °C in a freezer for further uses by being divided into small volumes. The desalted and concentrated dCas9 protein was run on the 10% sodium dodecyl sulfate-polyacrylamide gel electrophoresis (SDS-PAGE) gel and the existence of recombinant proteins dCas9-TADs with the anticipated molecular weight of ~160 kDa was confirmed (Supplementary Figure S5b).

### 4.9. Enzymes for protoplast isolation

#### 4.9.1. Enzyme Solution (Digestion Solution)

Enzyme solution was prepared by mixing different concentrations of cellulase R-10 and macerozyme R-10 (Table 3), 0.4 M D-mannitol, 20 mM MES (pH 5.7), 10 mM CaCl_2_, 20 mM KCl, and 0.1% (*w/v*) Bovine Serum Albumin (BSA). The enzyme mixture was warmed at 55 °C for 10 min to inactivate the functions of proteases and DNAse and cooled down to room temperature. After cooling down to room temperature it was sterilized by filtration utilizing a 0.22 µm filter. The enzyme solution was freshly prepared before being put to use.

#### 4.9.2. W5 Solution

To prepare W5 solution, 154 mM NaCl, 125 mM CaCl_2,_ 5 mM KCl and 2 mM MES (pH 5.7) were mixed and filter sterilization was performed by using a 0.22 µm filter. After filtration, the mixture was stored at 4 °C for later use.

#### 4.9.3. MMG Solution

MMG solution was prepared by combining 0.4 M D-mannitol, 15 mM MgCl_2_, and 4 mM MES (pH 5.7). The solution was mixed properly and sterilized by a 0.22 µm filter. After sterilization, the solution was kept at room temperature for further utilization.

#### 4.9.4. WI Solution

WI solution was formulated by using 0.5 M D-mannitol, 20 mM KCl and 4 mM MES (pH 6). The prepared solution was kept at room temperature by filter sterilization utilizing 0.22 µm filters.

#### 4.9.5. PEG-Calcium Transfection Solution

PEG-Calcium transfection solution was prepared by mixing 40% (*w/v*) PEG4000. 0.6 M D-mannitol and 100 mM CaCl_2_. The solution was prepared freshly just before utilization after sterilization through filtration by a 0.22 µm filter.

#### 4.9.6. FDA Solution

Five milligrams of fluorescein diacetate (FDA; Sigma-Aldrich) were dissolved in 1 mL of acetone to prepare FDA stock solution of 0.5% and kept at −20 °C in the dark.

### 4.10. Protoplast Isolation from Leaf Mesophyll and Callus Tissue

Five hundred milligrams of fully expanded upper leaves and callus were collected from 28 d-old plantlets grown on MS media and callus-inducing media, respectively. The harvested leaves and callus were cut into 0.5–1.0 mm strips by using a very sharp razor blade and shifted into a sterilized Petri dish containing 10 mLof freshly prepared enzyme solutions (digestion solution) containing different concentrations of cellulase R-10 and macerozyme R-10. The leaf strips were mixed properly and incubated for 16 h by gentle shaking at 50 rpm and kept at room temperature under dark conditions. After incubation, the digestion was postponed by adding an equal volume (10 mL) of W5 solution to the Petri dish, and handshaking was performed for 1 min. The entire mixture was filtered through a 120 µm nylon mesh to a 50 mLfalcon tube. This filtration process was allowed to remove the debris and undigested leaf tissues from the mixture. The digested mixture was centrifuged at 150× *g* for 5 min at room temperature. The supernatant was discarded carefully without disturbing the pellets of protoplasts. The harvested pellets were again dissolved in 10 mL of cold W5 solution and kept on ice for 30 min. After 30 min of incubation on ice, the mixture was again centrifuged at 150× *g* for 5 min at room temperature and the supernatant was discarded carefully without losing protoplasts. The pellets were gently re-dissolved in MMG solution to adjust the concentration at 4 × 10^5^ protoplasts per mL for further transfection.

### 4.11. Assessment of Protoplast Yield and Viability

A Leica DM2500 microscope (Leica, Wetzlar, Germany) was used to count and photograph the protoplasts, and a hemocytometer was used to calculate the approximate protoplast yield. The yield of protoplast was calculated as the total number of protoplasts released in the enzyme mixture divided by the fresh weight (g of FW) of the tissues used for protoplast isolation (no. of protoplasts/g of FW). Protoplast viability was estimated by using the FDA by following the protocol described elaborately before [54,55]. For staining the viable protoplast, 1 µL of FDA (0.5%) staining solution was mixed with 49 µL of isolated protoplast and kept at room temperature for 5 min. The viable protoplasts were stained as green fluorescence and visualized. The photograph was captured by using confocal microscopy. The viability was calculated as no. of green protoplasts divided by no. of total protoplasts and multiplied by 100%. Three images were captured for each sample and each experiment was repeated three times.

### 4.12. Ribonucleoprotein (RNP) Complexes Preparation

The following CRISPR/dCas9 ribonucleoproteins (RNPs) were employed in this study: dCas9-TAD proteins and in vitro-transcribed sgRNA18, sgRNA30, sgRNA33, and sgRNA34 were used to make RNP18, RNP30, RNP33, and RNP34 complexes, respectively. RNP complexes were made by gradually combining 25 µg (50 µL) of dCas9-TAD proteins with 25 µg (10 µL) of various sgRNAs, as discussed before [37,38]. To generate RNPs, they were incubated at room temperature for 10 min. Through PEG-mediated transfection, the resulting RNP complexes (20 µL) were directly delivered into 8 × 10^4^ (200 µL) stevia protoplasts.

### 4.13. PEG-Mediated Transfection of Protoplasts with dCas9-TADs RNP Complexes

Transfection with polyethylene glycol (PEG) was carried out similarly to the method previously described by Woo et al. (2015) [38]. Isolated protoplasts were transfected with pre-assembled RNP complexes to precisely stimulate the *UGT76G1* target gene in stevia. A mixture of 8 × 10^4^ protoplasts resuspended in MMG solution (200 µL) was gently mixed with 20 µL of RNP and 210 µL of freshly prepared PEG solution (40% (*w/v*) PEG 4000, 0.1 M CaCl_2_, and 0.2 M mannitol), and then incubated at 28 °C for 10 min in the dark. After incubation, 950 µL of W5 solution (2 mM MES (pH 5.7), 154 mM NaCl, 125 mM CaCl_2_, and 5 mM KCl) were gradually added by pipette into the reaction tube. By inverting the tube alternately, the solution was thoroughly mixed. Pelleted protoplasts were extracted by centrifugation at 100× *g* for 3 min, then slowly resuspended in 1 mLWI solution (4 mM MES (pH 5.7), 0.5 M mannitol, and 20 mM KCl). The protoplasts were incubated in Petri plates pre-coated with 1% BSA for 24 h at 28 °C in the darkness for CRISPR/dCas9 gene activation. Protoplasts were extracted a day after transfection for RNA isolation and a gene expression experiment was conducted to detect CRISPR/dCas9 gene activation. In the case of control protoplasts, transfection was carried out by using nuclease-free water instead of RNP.

### 4.14. RNA Extraction from Transfected Protoplasts and cDNA Synthesis

Total RNA was extracted from transfected protoplasts by using TRI Reagent^®^ (, Sigma Aldrich Chemie GmbH, Eschenstr. 5, 82024 Taufkirchen, Germany, Catalog Number: 9404) by following the manufacturer’s guidelines with some modifications. After incubation of a total of 8 × 10^4^ (200 µL) transfected stevia protoplasts with different RNPs in WI solution for 24 h at 28 °C were harvested by centrifuging at 150× *g* for 5 min. The harvested protoplasts were washed with PBS (pH 7.00) and aggregated by centrifugation at 150× *g* for 5 min. The supernatants were removed as much as possible without losing any protoplasts. The harvested protoplasts were flash frozen by using liquid nitrogen for 10 min. One milliliter TRI Reagent^®^ was added directly into the frozen protoplasts and mixed by repeated pipetting and allowed at room temperature for 5 min for complete dissociation after homogenization. Biotechnology-grade chloroform (200 µL) was added and vortexed vigorously for 15 s and incubated at room temperature for 15 min. After incubation, the samples were centrifuged at 12,000× *g* for 15 min at 4 °C. After centrifugation, the lysates were separated into three different layers and the upper clear layer was transferred into a new 1.5 mL Eppendorf tube without touching the middle layer by leaving around 20% of clear supernatant. A total of 500 µL of 2-isopropanol were added into the supernatants and mixed by pipetting and inverting the tube five times and incubating at room temperature for 10 min. The solution was centrifuged at 10,000× *g* for 10 min at 4 °C and the supernatants were discarded carefully to avoid loss of RNA pellets. The RNA pellets were washed twice with 75% ethanol prepared in DEPC-treated water and finally, the RNAs were harvested by centrifuging at 7500× *g* for 5 min at 4 °C. The harvested RNA pellets were air dried for 10 min at room temperature and dissolved in 62 µL nuclease free water and mixed by repeated pipetting. The dissolved RNAs were incubated at 55 °C for 5 min by a water block heater and vortexed briefly for the complete dissolution of RNA into nuclease-free water. The concentrations of RNAs were determined by using Thermo Scientific NanoDrop^TM^ 100 Light Spectrophotometer. 

The harvested RNAs were treated with DNase I (1 U) RNase-free enzyme (Thermo SCIENTIFIC) as per the recommendation of the manufacturer. The total RNA (162 to 670.5 ng per 20 µL) was reverse transcribed with SensiFAST™ cDNA Synthesis Kit (Bioline Ltd., UNITED KINGDOM, Catalog Number: BIO-65053) according to the manufacturer’s procedures.

### 4.15. qPCR Analysis

Gene-specific primers for targeted gene *UGT76G1* along with *UGT74G1* and *UGT85C2* and reference genes; *Actin* (AF548026.1), *Aquaporin* (DQ269455.1), and *Calmodulin* (AF474074.1) were designed and synthesized as listed in Table 4. A Bio-Rad CFX96 real-time machine (C1000 Touch thermal cycler) was used to perform quantitative real-time PCR with SensiFast SYBR No-ROX Kit (Bioline Ltd., UNITED KINGDOM, Catalog Number: BIO-98005). The reaction mixture containing the final concentrations of 1X SensiFast SYBR No-ROX, template cDNA (5 ng of total RNA), and 200 nM of each forward and reverse primer was prepared in a final volume of 10 µL. No template control (NTC) was prepared to contain all reaction mixtures without the template. NTC was set as negative control wherein the absence of amplification denoted no cross contamination mixtures. The qPCR was performed using samples of three pooled biological replications (control and each group of treatment) with three technical replicates. The qPCR analysis was performed with cycling parameters set up as in Table 5. The expression patterns of each targeted gene were normalized to three reference genes according to the Livak method [91] using the Bio-Rad CFX Manager^TM^ software, version 3.1 (Bio-Rad, 1000 Alfred Nobel Drive, CA, USA).

### 4.16. Statistical Analysis

The Statistical Analysis System (SAS) program (version 9.4) was used to perform statistical analysis. All values were shown as mean ± SE (Standard Error); (*n* = 3) represented the means for three biological replications per treatment. Data were subjected to a one-way analysis of variance (ANOVA) for mean comparison and significant differences were calculated according to the Student’s t-test. The probability level for all statistical analyses was 0.05.

## 5. Conclusions

This study has established a platform for transcriptional activation of *UGT76G1* in protoplasts of stevia. Utilizing PEG-mediated transfection of protoplasts with RNPs, we exhibited successful transcriptional activation. The technique which employs CRISPR/dCas9 offers several advantages including simplicity in execution, rapidity in generating results, eradication of transgene integration, and minor DNA insertions in mutants. The stevia protoplast having transcriptionally activated *UGT76G1* with CRISPR/dCas9 RNPs lacking integrated transgenes, their deployment in practical breeding and commercialization should be more publicly acceptable, expediting precision agriculture development. Furthermore, the regenerated plants from protoplast might be exempt from the prevailed GMO regulations because the recombinant DNA was not used in this platform. This technique may open the way for increased adoption of valuable crops generated using this precision biotechnology. More research is needed to maximize plant regeneration from CRISPR RNPs converted protoplasts in order to investigate the field uses of this technology.

## Figures and Tables

**Table 1:** Designed sgRNAs targetingthe *UGT76G1* gene. RNAfold web server was used in determining the secondary structure and other features (loops and base pairing).

sgRNA	Strand	Secondary Structure (Vienna Format)	TSL	CBP	TBP	IBP
4	+	UGGAAAAUGCACAUCUUGUGGUUUUAGAGCUAGAAAUAGCAAGUUAAAAU AAGGCUAGUCCGUUAUCAACUUGAAAAAGUGGCACCGAGUCGGUGCUUUU ..((.((((.((.((((((.........((((....))))........))))))...)).)))).)).((((....))))(((((((...)))))))...	5	6	14	0
13	−	UUAUGGGGAACUUUUUUGCAGUUUUAGAGCUAGAAAUAGCAAGUUAAAAU AAGGCUAGUCCGUUAUCAACUUGAAAAAGUGGCACCGAGUCGGUGCUUUU .........(((((((((.((((....(((..((..((((.............)))).)).)))...)))))))))))))(((((((...)))))))...	4	9	10	0
18	−	AACUAAGGUAGUAAGGCAAAGUUUUAGAGCUAGAAAUAGCAAGUUAAAAU AAGGCUAGUCCGUUAUCAACUUGAAAAAGUGGCACCGAGUCGGUGCUUUU .((((...))))..(((...(((((((.((((....))))...)))))))...)))............((((....))))(((((((...)))))))...	3	4	7	4
25	+	AUUGUUGAACUCGAUUAGGGGUUUUAGAGCUAGAAAUAGCAAGUUAAAAU AAGGCUAGUCCGUUAUCAACUUGAAAAAGUGGCACCGAGUCGGUGCUUUU ...(((((...(((((((..(((((((.((((....))))...)))))))....))))).))...)))))..........(((((((...)))))))...	4	7	12	0
26	+	CUCGAUUAGGGCGGCCCAAGGUUUUAGAGCUAGAAAUAGCAAGUUAAAAU AAGGCUAGUCCGUUAUCAACUUGAAAAAGUGGCACCGAGUCGGUGCUUUU ...(((..((((((((....(((((((.((((....))))...)))))))..)))).))))...))).((((....))))(((((((...)))))))...	4	8	11	0
29	+	UUAGGGCGGCCCAAGAGGUAGUUUUAGAGCUAGAAAUAGCAAGUUAAAAU AAGGCUAGUCCGUUAUCAACUUGAAAAAGUGGCACCGAGUCGGUGCUUUU ...((((((((.........(((((((.((((....))))...)))))))..)))).)))).......((((....))))(((((((...)))))))...	3	8	8	0
30	−	GGGGCCUAAAGCACAAGCUUGUUUUAGAGCUAGAAAUAGCAAGUUAAAAU AAGGCUAGUCCGUUAUCAACUUGAAAAAGUGGCACCGAGUCGGUGCUUUU .((((....(((.....((((((((((.((((....))))...))))))))))))).)))).......((((....))))(((((((...)))))))...	3	4	10	0
33	−	AAGGGGGCUGUAUAGAAGUGGUUUUAGAGCUAGAAAUAGCAAGUUAAAAU AAGGCUAGUCCGUUAUCAACUUGAAAAAGUGGCACCGAGUCGGUGCUUUU .((.(((((....((.....(((((((.((((....))))...)))))))....))))))).))....((((....))))(((((((...)))))))...	4	6	10	0
34	−	AAAGGGGGCUGUAUAGAAGUGUUUUAGAGCUAGAAAUAGCAAGUUAAAAU AAGGCUAGUCCGUUAUCAACUUGAAAAAGUGGCACCGAGUCGGUGCUUUU ..((.(((((....((...((((((((.((((....))))...))))))))...))))))).))....((((....))))(((((((...)))))))...	4	5	10	0
35	−	AAAAGGGGGCUGUAUAGAAGGUUUUAGAGCUAGAAAUAGCAAGUUAAAAU AAGGCUAGUCCGUUAUCAACUUGAAAAAGUGGCACCGAGUCGGUGCUUUU ...((.(((((((.......(((((((.((((....))))...)))))))...)).))))).))....((((....))))(((((((...)))))))...	3	7	9	0

**Table 2:** Yield and purity of RNAs extracted from transfected protoplasts of stevia.

RNA	Before DNAse I Treatment		After DNAse I Treatment	
	Yield (ng/µL)	Purity (A_260_/A_280_)	Yield (ng/µL)	Purity (A_260_/A_280_)
Control	59.6	1.62	44.7	1.77
RNP18	12.2	1.72	11.1	1.72
RNP30	15.4	1.77	14.2	1.77
RNP33	12.2	1.79	10.8	1.80
RNP34	14.4	1.79	12.5	1.80

**Table 3:** Different concentrations of cellulase R-10 and macerozyme R-10 for the preparation of the enzyme solutions.

Enzyme Solution	Cellulase R-10 (%)	Macerozyme R-10 (%)
ES1	1.00	0.50
ES2	1.00	0.75
ES3	1.00	1.00
ES4	1.25	0.50
ES5	1.25	0.75
ES6	1.25	1.00
ES7	1.50	0.50
ES8	1.50	0.75
ES9	1.50	1.00

**Table 4:** Details of the designed primers used to detect the expression level of targeted and reference genes in qPCR.

Name (Accession No.)	Forward (F)/ Reverse (R)	Sequence (5′—3′)	Amplicon Size (bp)	Annealing Temperature (°C)
*Actin* (AF548026.1)	F	CTGAGAACTGAGGGCTAGGG	187	70.5
	R	AACCCAGCCTTGACCATTCC		
*Aquaporin* (DQ269455.1)	F	GGAGCCGCCGTAATCTACAA	86	80
	R	GCAATCGCCGCACCAATAAA		
*Calmodulin* (AF474074.1)	F	ATCCGCTCACCGACGATCA	142	65
	R	TGCAGTTCAGCTTCTGTTGG		
*UGT76G1*(KM206772.1)	F	CTGCCAATGCCACCGTTATT	94	52
	R	TCATAAACCGTCTGACGCAGG		
*UGT74G1*(AY345982.1)	F	TTCCAGTGCTTCAACGGTGG	124	61
	R	GTGAAGACCCAACGTGCTTG		
*UGT85C2*(AY345978.1)	F	CGTTCGATGAGTTGGAGCCT	181	61
	R	AGCCACTGGAAACACTCTGG		

**Table 5:** Cycling parameters for qPCR.

Step	Temperature	Duration	Cycle (s)
Initial activation	95 °C	2 Min.	1
Denaturation	95 °C	05 S	40
Annealing	* 52–80 °C	10 S	
Extention	72 °C	15 S	
Melt curve	65–95 °C	5 S for every increment of 0.5 °C	

* the optimized annealing temperature for each targeted and reference gene was set accordingly.

## Data Availability

Not applicable.

## Supplementary Materials

The following supporting information can be downloaded at: https://www.mdpi.com/article/10.3390/plants11182393/s1. Figure S1: Microscopic view of protoplasts isolated from stevia. Scale bar: 50 µm; Figure S2: Total RNAs isolated from stevia protoplasts transfected with RNP complex containing different sgRNAs and the RT-PCR products of these RNAs. The total RNAs were isolated 24 h after transfection and used in reverse transcription using UGT76G1-specific primers. (a) Isolated total RNAs from transfected protoplasts; and (b) RT-PCR products using these RNAs; Figure S3: The amplified stevia UGT76G1 promoter fragment with 5′-UTR sequence. Genomic DNA isolated from stevia was used as a template in PCR amplification using gene-specific primers flanking the reported promoter region (KM206772.1) and the PCR product was sequenced. Softberry (http://www.softberry.com/berry.phtml?topic=tssp&group=programs&subgroup=promoter, accessed on 27 April 2021) was used to determine the positions of the TSS and TATA box: (a) Agarose gel electrophoresis of the amplified PCR product Lane M: DNA ladder; 1: first replication; 2: second replication; and (b) Complete sequence ranges from −529 to 1309 nucleotide position (1839 bp) with 99% identity of KM206772.1 and yellow boxes showing the positions of transcription start site (TSS) at +1 and the TATA box at −33 nucleotide positions. The region from −1 to −529 represents the promoter region and the region from 2 to 1309 represents 5′ UTR; Figure S4: Quality of the in vitro transcribed sgRNAs targeting UGT76G1. The sgRNA-DNA templates were PCR assembled and used in in vitro transcription reactions to synthesize the sgRNAs. The DNA templates produced by PCR and the synthesized sgRNAs were analyzed by agarose gel electrophoresis: (a) The assembled sgRNAs-DNA template sequences for the different sgRNAs; blue, red, and green-colored text represents T7 primer sequences, sgRNA sequences, and crRNA/tracrRNA 80-nt constant region, respectively; (b) sgRNAs-DNA templates produced by PCR; and (c) In vitro transcribed sgRNAs; and Figure S5: dCas9-VP64 fusion protein over-expressed in Rosetta (DE3) E. coli: (a) Schematic diagram of pET-dCas9-VP64-6xHis vector used for cloning in E. coli; and (b) Over-expressed dCas9-VP64 fu-sion protein analyzed by 10% SDS-PAGE.
