# Supplementary material for: DNA Free CRISPR/DCAS9 Based Transcriptional Activation System for UGT76G1 Gene in Stevia rebaudiana Bertoni Protoplasts
Source: Plants (Basel). 2022 Sep 14;11(18):2393. doi: 10.3390/plants11182393 (PMC9501275; doi:10.3390/plants11182393)
Supplement: Supplementary file 1 [file plants-11-02393-s001.zip › Supplementary Figure S2.pdf]

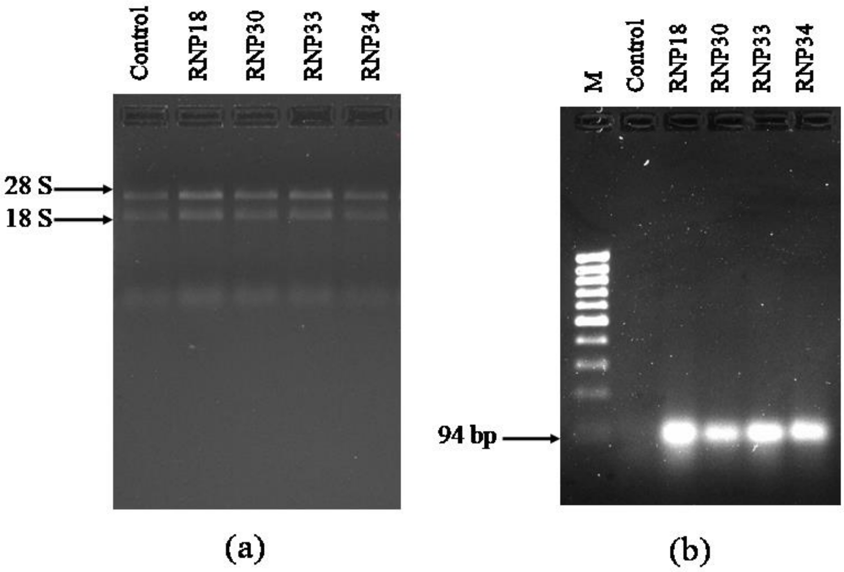

Supplementary Figure S2. Total RNAs isolated from stevia protoplasts transfected with RNP complex containing different sgRNAs and the RT-PCR products of these RNAs. The total RNAs were isolated 24 h after transfection and used in reverse transcription using *UGT76G1*-specific primers. **(a)** Isolated total RNAs from transfected protoplasts; and **(b)** RT-PCR products using these RNAs.
