# Supplementary material for: DNA Free CRISPR/DCAS9 Based Transcriptional Activation System for UGT76G1 Gene in Stevia rebaudiana Bertoni Protoplasts
Source: Plants (Basel). 2022 Sep 14;11(18):2393. doi: 10.3390/plants11182393 (PMC9501275; doi:10.3390/plants11182393)
Supplement: Supplementary file 1 [file plants-11-02393-s001.zip › Supplementary Figure S3.pdf]

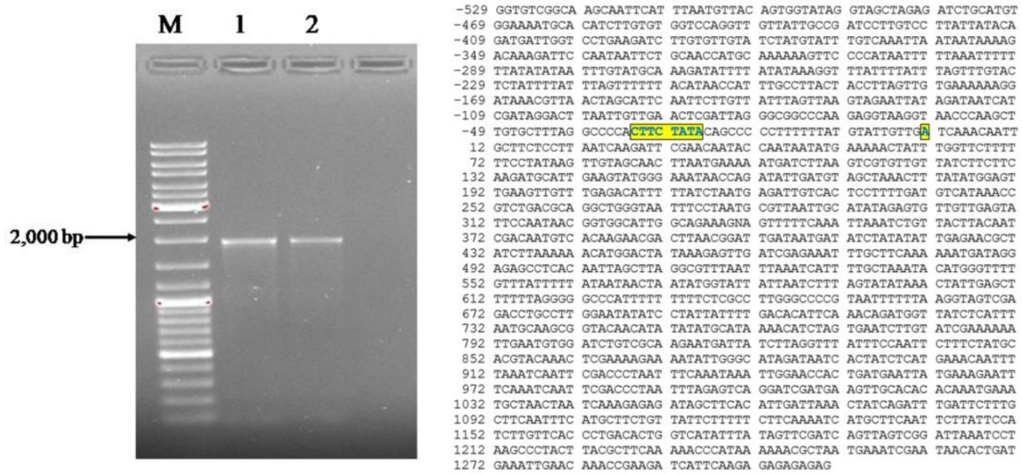

(a) (b)

Supplementary Figure S3. The amplified stevia UGT76G1 promoter fragment with 5′-UTR sequence. Genomic DNA isolated from stevia was used as a template in PCR amplification using gene-specific primers flanking the reported promoter region (*KM206772.1*) and the PCR product was sequenced. Softberry (http://www.softberry.com/berry.phtml?topic=tssp&group=programs&subgroup=promoter accessed on 27 April, 2021) was used to determine the positions of the TSS and TATA box: (**a**) Agarose gel electrophoresis of the amplified PCR product Lane M: DNA ladder; 1: first replication; 2: second replication; and (**b**) Complete sequence ranges from -529 to 1,309 nucleotide position (1,839 bp) with 99% identity of *KM206772.1* and yellow boxes showing the positions of transcription start site (TSS) at +1 and the TATA box at -33 nucleotide positions. The region from -1 to -529 represents the promoter region and the region from 2 to 1,309 represents 5′ UTR.
