# Supplementary material for: DNA Free CRISPR/DCAS9 Based Transcriptional Activation System for UGT76G1 Gene in Stevia rebaudiana Bertoni Protoplasts
Source: Plants (Basel). 2022 Sep 14;11(18):2393. doi: 10.3390/plants11182393 (PMC9501275; doi:10.3390/plants11182393)
Supplement: Supplementary file 1 [file plants-11-02393-s001.zip › Supplementary Figure S4.pdf]

sgRNA18	5′TAATACGACTCACTATAG**AACTAAGGTAGTAAGGCAAA**GTT TTAGAGCTAGAAATAGCAAGTTAAAATAAGGCTAGTCCGTTAT CAACTTGAAAAAGTGGCACCGAGTCGGTGCTTTT-3′
sgRNA30	5′TAATACGACTCACTATAG**GGGGCCTAAAGCACAAGCTT**GTT TTAGAGCTAGAAATAGCAAGTTAAAATAAGGCTAGTCCGTTAT CAACTTGAAAAAGTGGCACCGAGTCGGTGCTTTT-3′
sgRNA33	5′TAATACGACTCACTATAG**AAGGGGGCTGTATAGAAGTG**GTT TTAGAGCTAGAAATAGCAAGTTAAAATAAGGCTAGTCCGTTAT CAACTTGAAAAAGTGGCACCGAGTCGGTGCTTTT-3′
sgRNA34	5′TAATACGACTCACTATAG**AAAGGGGGCTGTATAGAAGT**GTT TTAGAGCTAGAAATAGCAAGTTAAAATAAGGCTAGTCCGTTAT CAACTTGAAAAAGTGGCACCGAGTCGGTGCTTTT-3′

(a)

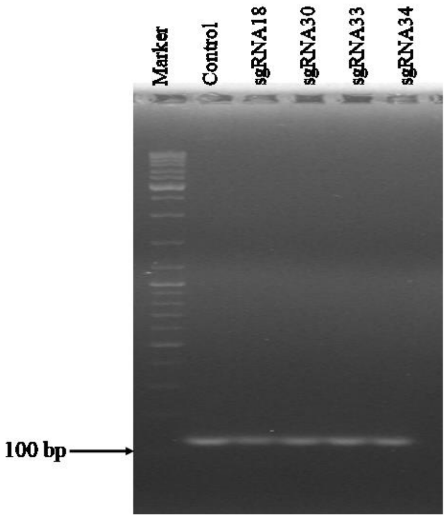

(b)

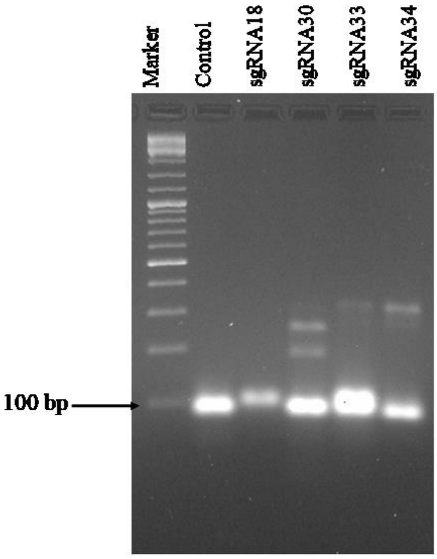

(c)

Supplementary Figure S4. Quality of the *in vitro* transcribed sgRNAs targeting *UGT76G1*. The sgRNA-DNA templates were PCR assembled and used in *in vitro* transcription reactions to synthesize the sgRNAs. The DNA templates produced by PCR and the synthesized sgRNAs were analyzed by agarose gel electrophoresis: **(a)** The assembled sgRNAs-DNA template sequences for the different sgRNAs; blue, red, and green-colored text represents T7 primer sequences, sgRNA sequences, and crRNA/tracrRNA 80-nt constant region, respectively; **(b)** sgRNAs-DNA templates produced by PCR; and **(c)** *In vitro* transcribed sgRNAs
