# Supplementary material for: DNA Free CRISPR/DCAS9 Based Transcriptional Activation System for UGT76G1 Gene in Stevia rebaudiana Bertoni Protoplasts
Source: Plants (Basel). 2022 Sep 14;11(18):2393. doi: 10.3390/plants11182393 (PMC9501275; doi:10.3390/plants11182393)
Supplement: Supplementary file 1 [file plants-11-02393-s001.zip › Supplementary Figure S5.pdf]

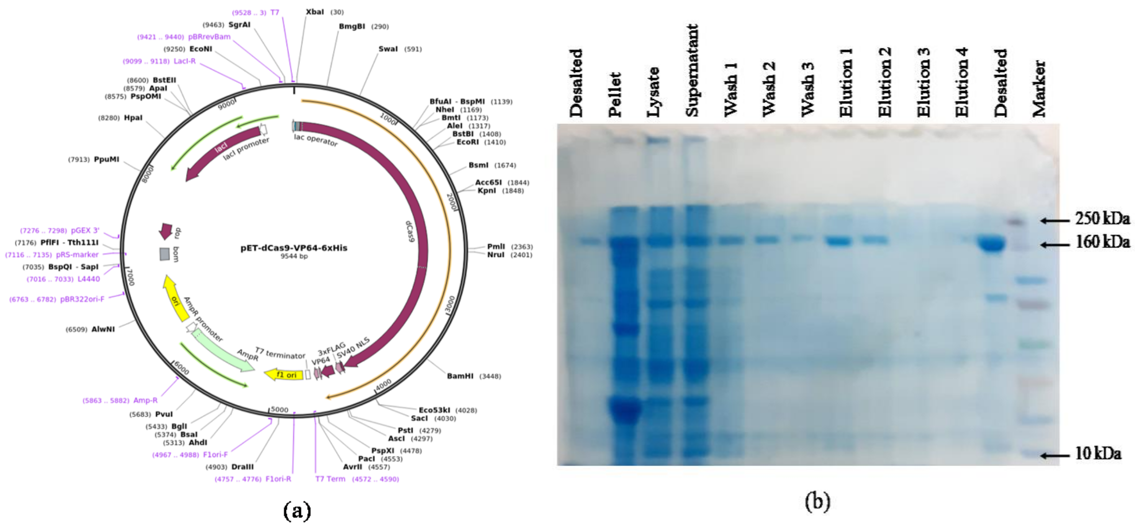

Supplementary Figure S5. dCas9-VP64 fusion protein over-expressed in Rosetta (DE3) *E. coli:* **(a)** Schematic diagram of pET-dCas9-VP64-6xHis vector used for cloning in *E. coli*; and **(b)** Over-expressed dCas9-VP64 fusion protein analyzed by 10% SDS-PAGE.
