# Supplementary figures and images for: DNA Free CRISPR/DCAS9 Based Transcriptional Activation System for UGT76G1 Gene in Stevia rebaudiana Bertoni Protoplasts
Source: Plants (Basel). 2022 Sep 14;11(18):2393. doi: 10.3390/plants11182393 (PMC9501275; doi:10.3390/plants11182393)

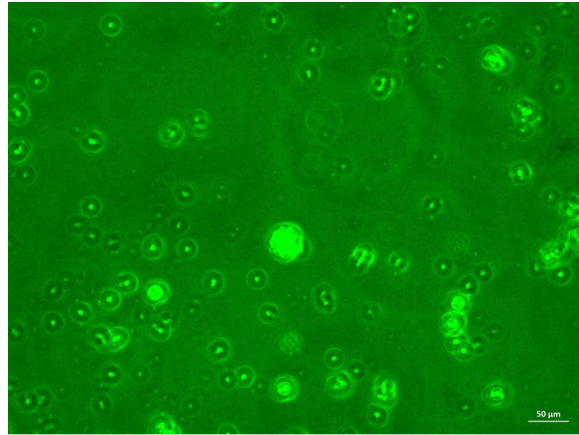

**Supplementary Figure S1.** Microscopic view of protoplasts isolated from stevia. Scale bar: 50 μm.

Supplement: Supplementary file 1 [file plants-11-02393-s001.zip › Supplementary Figure S1.pdf]
